# Motor performance in chronic low back pain: is there an influence of pain-related cognitions? A pilot study

**DOI:** 10.1186/1471-2474-12-211

**Published:** 2011-09-27

**Authors:** Dymphy Kusters, Miriam M Vollenbroek-Hutten, Hermie J Hermens

**Affiliations:** 1 Roessingh Research and Development, Enschede, The Netherlands; 2 Faculty of Electrical Engineering, Mathematics & Informatics, University of Twente, Enschede, The Netherlands

**Keywords:** chronic low back pain, movement speed, reaction time, pain-related cognitions

## Abstract

**Background:**

Chronic low back pain (CLBP) is often accompanied by an abnormal motor performance. However, it has not been clarified yet whether these deviations also occur during motor tasks not involving the back and whether the performance is influenced by pain and pain-related cognitions. Therefore, the aim of the present study is to get insight in the contribution of both pain experience and pain-related cognitions to general motor task performance in CLBP.

**Methods:**

13 CLBP patients and 15 healthy subjects performed a hand-function task in three conditions: sitting, lying prone (lying) and lying prone without trunk support (provoking). The last condition was assumed to provoke pain-related cognitions, which was considered successful when a patients' pain expectancy on a numeric rating scale was at least 1 point higher than actual pain experienced. Subjects' performance was expressed in reaction time and movement time. Repeated measures analysis of variance was performed to detect main effect for group and condition. Special interest was given to group*condition interaction, since significant interaction would indicate that patients and healthy subjects performed differently throughout the three conditions.

**Results:**

Patients were slower throughout all conditions compared to healthy subjects. With respect to the provoking condition, patients showed deteriorated performance compared to lying while healthy subjects' performance remained equal between these two conditions. Further analysis of patients' data showed that provocation was successful in 54% of the patients. Especially this group showed deteriorated performance in the provoking condition.

**Conclusion:**

It can be concluded that CLBP patients in general have worse motor task performance compared to healthy subjects and that provoking pain-related cognitions further worsened performance.

## Background

Chronic low back pain (CLBP) is often accompanied by deviations in motor performance [1-3]. Frequently, these deviations are attributed to physical adaptations to pain as described in the pain-adaptation model [4]. However, other studies suggest that deviated motor performance might better be explained by the influence of pain-related cognitions [5,6]. According to the fear-avoidance model the relation between pain and motor performance is characterized by a negative vicious circle of avoidance behavior and increased pain that is preserved by fear [6]. Indeed, various studies confirm that CLBP patients scoring high on kinesiophobia perform motor tasks worse than their less fearful counterparts [7-9].

However, to date the majority of research on motor performance in CLBP has been focusing only on motor tasks directly involving the lower back (e.g. reaching, bending). As these lower back motor tasks are often considered both painful and threatening by patients, it is hard to distinguish whether deviations in performance should be attributed to pain experience or to pain-related cognitions. Although insight in the effect of these parameters separately would provide valuable information regarding the CLBP problem, only a few studies tried to enable analysis of pain effect apart from cognition effect. Lamoth and coworkers studied the influence of both parameters on gait in healthy subjects [10]. Their results show that pain induction has an influence on gait parameters, while induction of fear of pain had not. However, subjects knew that pain would disappear eventually and, thus, their pain-related fear might not be representative for a chronic pain population where pain is present continuously and pain-related fear is much more substantial. Pfingsten and colleagues studied the effect of pain-related cognitions on performance of a leg flexion task in CLBP patients [11]. Cognitions were manipulated by telling the experimental group that the task was potentially painful, while the control group was reassured that no harm or pain would occur. Their results indicate that the experimental group showed worse task performance. However, besides fear also pain experience was increased in the experimental group. Therefore, neither the effect of pain experience nor that of pain-related fear could be individually distinguished.

In sum, there is lack of knowledge concerning the relation between pain, pain-related cognitions and deviations in CLBP patients' motor performance. As it is conceivable that managing pain demands a different treatment approach than managing pain-related cognitions, this knowledge might be useful to increase CLBP therapy effectiveness.

Therefore, the aim of the present study is to gain insight in motor performance of CLBP as well as the contribution of both pain experience and pain-related cognitions to motor task performance. For this, both CLBP patients and healthy subjects were asked to perform a hand-function task in a neutral sitting position and two conditions (lying and provoking) that were assumed to increasingly provoke pain-related cognitions.

It was hypothesized that patients would perform worse compared to healthy subjects in all conditions. Furthermore, it was expected that performance would worsen from neutral to lying condition in both groups and that performance in the provoking condition would be further deteriorated in CLBP patients, but not in healthy subjects.

## Methods

### Subjects

Fifteen CLBP patients and fifteen age-matched subjects without low back complaints participated in this study. Patients were referred from the Roessingh Rehabilitation Centre and local physiotherapists. Healthy subjects were recruited amongst family and friends of Roessingh Research and Development personnel and by poster advertising in local (sports) clubs.

To be included patients had to be at least 18 years of age, have chronic low back pain without identifiable pathological causes, continuously or recurrently present for at least 12 weeks, and be free of other pain problems. As the objective of the study was to gain insight in the contribution of both pain experience and pain-related cognitions to motor task performance in CLBP patients, patients that were not experiencing pain at the time of testing were excluded from participation. Healthy subjects were included if they were least 18 years of age and had not had (back) pain complaints during at least 6 months previous to study participation. Both patients and healthy subjects were excluded from the study in case of upper-extremity disorders, psychopathology or insufficient mastering of the Dutch language. In addition, experimental layout required subjects' heights to be between 150 cm and 200 cm. Consequently, subjects that did not meet this criterion were also excluded from the study.

All included subjects gave their written informed consent prior to participation in this study. The study protocol was approved by the local Medical Ethics Committee.

### Questionnaires

All participants were asked to fill out a question form regarding socio-demographic characteristics (age, gender, height, handedness and level of education). In addition, CLBP patients completed a series of self-report questionnaires to assess pain intensity, fear of movement and pain catastrophizing.

Pain intensity was measured by a visual analogue scale (VAS). Patients were asked to score their minimal and maximal pain in the last week and present pain on 100 mm lines anchored on the left with "no pain at all" and on the right with "worst pain imaginable".

Fear of movement was assessed by the Dutch version of the TAMPA scale of kinesiophobia (TSK-DV [6]), a 17-item questionnaire to measure patients' fear of pain due to movement. At each item, patients had to score their agreement on a 4-point Likert scale with scoring alternatives ranging from "strongly disagree" to "strongly agree". After inversion of the scores on items 4, 8, 12 and 16, a total score was calculated. Higher scores reflect a higher fear of movement.

To assess level of pain catastrophizing the Dutch version of the Pain Catastrophizing Scale (PCS-DV [12]) was used. This questionnaire comprises 13 items concerning patients' beliefs and feelings regarding their pain. For each item patients scored how frequently the described thought occurred to them on a 5-point scale ranging from 0 (= never) to 4 (= always). Afterwards, the total score was calculated. Higher scores indicate a higher level of pain catastrophizing.

### Experimental protocol

Subjects had to perform a motor task based on the hand-function task described by Jebsen et al. [13]. The task required subjects to respond to an auditory signal by moving five cylinders as fast as possible from the back to the front of a table and vice versa. Subjects were instructed to push a button with their dominant hand until they heard a beep. After hearing the beep, they had to release the button and start moving the cylinders in a specified order; first all cylinders had to be moved from the back to the front of the table, than all cylinders had to be moved back. Also cylinder movement had to be performed with the subject's dominant hand. In the instruction it was emphasized that the complete task had to be performed as fast as possible.

To rule out learning, the actual test phase was preceded by a practice phase. It was assumed that subjects had mastered the test sufficiently if they were equally fast (time difference less than 500 ms) in three subsequent trials.

**Figure 1:**
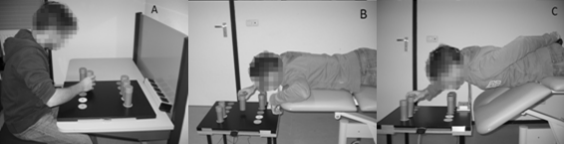
**Experimental conditions of task performance**. A) Sitting condition; B) Lying condition; C) Provoking condition.

After this practice phase the actual test phase began. The motor task was performed subsequently in the three different conditions illustrated in Figure 1: sitting in a chair - being a neutral, familiar position and, therefore, not assumed to provoke pain or cognitions in any way - (sitting), lying prone on an examining table (lying) and lying prone without trunk support (provoking). In each condition the task was performed three times, with a short period of rest between conditions.

Especially, the provoking condition required active, volitional exertion of the back muscles and was, therefore, expected to provoke pain-related cognitions in the CLBP patients but not in the healthy subjects. To further increase the provoking effect of this condition, subjects were told that the posture was very demanding on their low back muscles. Provocation of pain cognitions was assumed to be successful if expected pain was higher than the pain patients actually experienced due to task performance. This assumption was based on the findings of a previous study by Crombez et al. [14]. To determine this, prior to each condition subjects were asked to score the pain they expected due to task performance on a numerical rating scale (NRS) ranging from 0 to 10, where 0 represented "no pain at all" and 10 represented "worst pain imaginable". After completion of the task in the particular condition subjects were asked to score the pain they had experienced due to task performance on another NRS. Successful manipulation was defined by NRS_expected _being at least 1 point higher than NRS_experienced_.

### Data collection

The auditory signal that subjects had to react to was generated by a random beep generator at a time interval ranging from 1 to 10 seconds after pressing the button. The interval was randomized to prevent subjects from anticipating the sound signal. Both onset of the auditory signal and release of the button were registered by means of an alteration to the output signal.

Light sensors were used to monitor cylinder placement. Sensor output signal altered in case of cylinder placement. All sensors generated a slightly different output signal to be able to distinguish separate cylinder placements. All signals were measured at an auxiliary channel of a TMSi REFA data acquisition device at a sampling frequency of 2048 Hz, using Portilab 2 software (TMS International, Oldenzaal, The Netherlands). Raw data were stored for off-line data analysis.

### Data analysis

To facilitate data analysis, the raw output signal was converted into time measures using a custom-written Labview routine (National Instruments, Austin, Texas, USA). After this final data processing stage, reaction time (RT) and movement time (MT) were determined. RT was defined as the time between onset of the auditory signal and release of the button. Minimal RT to an auditory stimulus (i.e. the minimal amount of time needed for registration of an auditory signal and a subsequent motor reaction) has proven to be between 140-160 ms in adults [15]. Therefore, in this study any RT measuring less than 140 ms was considered a measurement error and, consequently, was excluded from further data analysis. This applied to only three trials in the whole data set (< 1%). Per condition, the mean RT over three trials was selected for statistical analysis. In addition to RT, motor performance was represented by MT, which was defined as the time between picking up the first cylinder and putting down the last cylinder. Contrary to RT, there was no physiological limit to MT and, thus, no trials were excluded from further analysis. The mean MT over three trials was used for statistical analysis.

### Statistical analysis

In order to ensure that no socio-demographic differences existed between groups characteristics of CLBP patients and healthy subjects were analyzed by independent t-tests for numerical variables and by χ^2 ^tests for categorical variables.

A repeated measures analysis of variance was performed for analysis of both RT and MT. To evaluate group and condition effects post-hoc Sidak tests were performed. Special interest was given to group*condition interaction, since significant interaction would indicate that patients and healthy subjects performed differently throughout the three conditions.

Furthermore, relations between pain measures, baseline cognition measures (i.e. fear of movement and catastrophizing) and task performance were examined by using Pearson's correlation. Special attention was given to the relation between NRS scores and cognition measures as a significant relation would indicate mediating effect on performance.

## Results

Fifteen CLBP patients were originally included in the study. Yet two of them were excluded after questionnaire assessment, because their VAS scores appeared to be less than 10 mm, indicating no pain experience at the time of testing. The socio-demographic characteristics of the remaining 13 CLBP patients and those of the 15 healthy subjects are depicted in Table 1. Statistical analysis showed no differences between the two groups regarding these socio-demographic characteristics. The patient group was further characterized by moderate level of pain experience (VAS = 49 mm), rather high level of kinesiophobia (TSK = 40.2) [6] and moderate level of pain catastrophizing (PCS = 21.5) [12].

**Table 1:** Baseline characteristics of the study population

	Patients (n = 13)	Controls (n = 15)	Statistics
Age (yrs)	56.5 (9.7)	52.9 (12.6)	*t = 0.835 n.s*.

Length (cm)	172.6 (9.3)	176.5 (11.0)	*t = -0.990 n.s*.

Male/Female	7/6	6/9	*Χ^2^= 0.537 n.s*.

Left-/Right-handed	1/12	2/13	*Χ^2^= 0.232 n.s*.

Education (0-7 numerical scale)	4.2 (1.7)	5.5 (1.4)	*t = -2.042 n.s*

Pain related scores			

- *VAS*	49 (26)		

- *TSK*	40.2 (7.3)		

- *PCS*	21.5 (10.8)		

Table displays means (SD); VAS = actual pain experience, visual analogue scale; TSK = Tampa scale of kinesiophobia; PCS = Pain Catastrophizing Scale

With respect to the manipulation of pain-related cognitions, results showed that healthy subjects did not report either experienced pain (NRS_experienced _= 0) or expected pain (NRS_expected _= 0) in any of the three conditions. Contrarily, manipulation of pain-related cognition (i.e. overestimation of pain) by the provoking posture seemed successful in the patient group.

**Figure 2:**
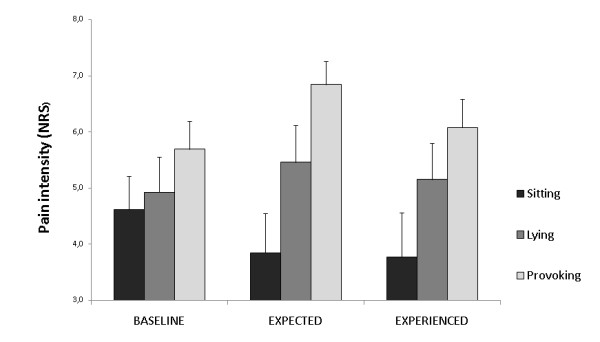
**Patients' pain ratings regarding task performance in the three experimental conditions**. NRS = numerical rating scale.

At group level (Figure 2) patients' baseline pain level appeared to increase slightly across conditions and this trend bordered significance (p = 0.064). With respect to experienced pain due to task performance NRS scores differed between sitting and both lying (p = 0.048) and provoking condition (p = 0.023), but there was no difference between lying and provoking condition (p = 0.226).

Furthermore, the hypothesis that the provoking condition would result in an increase in patients' expected pain compared to the experienced pain (i.e. provoke pain-related cognitions) was tested. Indeed, there was a trend of expected pain being higher than experienced pain, but this difference failed to reach significance (p = 0.175). Yet an explorative analysis on the individual patient scores showed that individual subjects reacted differently to the provocation. In seven out of 13 patients provocation appeared to be successful as they predicted higher expected pain than actual pain experience (i.e. NRS_expected _≥ NRS_experienced _+ 1). Contrarily, the other six patients predicted their pain correctly and, thus, seemed to be unsusceptible for the provoking condition. For this reason, it was decided to divide the patient population in two groups ("susceptible" vs. "unsusceptible") and perform separate analysis on these groups - additional to the analysis of group (patients vs. controls) and conditions effect - to explore whether differences in outcome measures could be attributed to susceptibility for provocation.

Demographic data of the two subgroups show no differences between the two groups, except for age: Susceptible patients were significantly older (mean (SD): 61.7 (6.4)) than the unsusceptible ones (mean (SD): 50.3 (9.5)). Therefore, in the statistical analysis of performance differences at subgroup level age was included as a covariate (ANCOVA). Interestingly, there appeared to be a trend that baseline pain (VAS) was lower in the susceptible patients than in the unsusceptible patients.

### Reaction time

**Figure 3:**
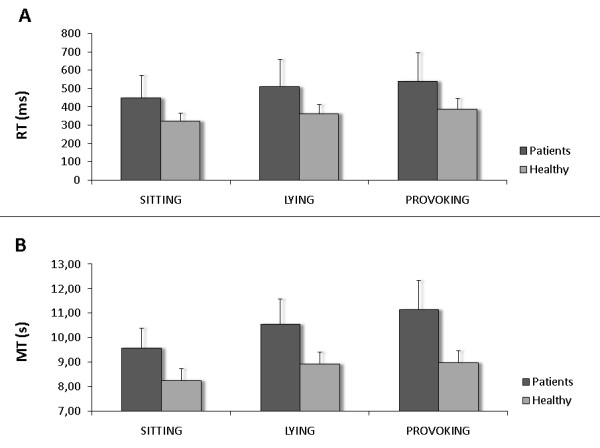
**Task performance of CLBP patients vs. healthy subjects across conditions**. A: Reaction times (RT); B: Movement times (MT); Error bars represent standard error of the mean (SEM).

Figure 3a presents the results of the repeated measures analysis of variance on RT. It appeared that CLBP patients showed slower RTs over all conditions compared to healthy subject. This was confirmed by a significant difference in overall RT (i.e. RT resumed over all conditions) between groups (p = 0.047).

Furthermore, condition appeared to have a significant effect on RT (p = 0.003). Both CLBP patients and healthy subjects showed increasing RTs across the three successive conditions. Post hoc Sidak testing showed that the condition effect was based on a significant difference between sitting compared to both lying and provoking condition (resp. p = 0.017 and p = 0.018), but not between lying and provoking condition (p = 0.559).

The increase in RT across conditions was not different between patients and healthy subjects as can be concluded from the lack of significant interaction between the three task conditions and experimental group (p = 0.804).

Results of the CLBP patients were additionally analyzed on differences between the two subgroups but this analysis revealed no differences with respect to RT between the two subgroups.

Correlation analysis showed only poor correlation of RT with NRS scores (r = - 0.032 to - 0.140), fear of movement (TSK; r = - 0.059 to - 0.363) and catastrophizing (PCS, r = 0.022 to 0.339) in all three task conditions. None of the correlations was significant at a P < 0.05 level. In addition, there were no significant correlations between NRS scores and TSK (r = - 0.204 to - 0.296; p > 0.3) or PCS (r = 0.152 to 218; p > 0.4).

### Movement time

The results of the repeated measures analysis of variance on MT are presented in Figure 3b. It clearly shows that patients were slower than healthy subjects over all conditions, which was proved by a significant group effect (p = 0.002).

**Figure 4:**
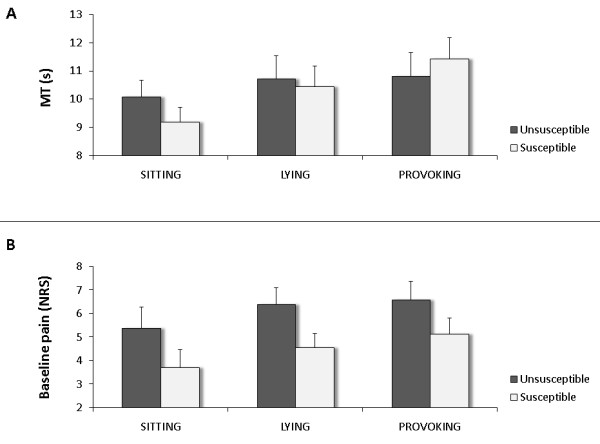
**Differences in performance across conditions between patients susceptible and unsusceptible to provocation corrected for age difference**. A: Movement times (MT) across conditions; B: Baseline pain ratings across conditions on a numerical rating scale (NRS); Error bars represent standard error of the mean (SEM).

Also the condition in which the task was performed affected MT (p < 0.001). Post hoc analysis (Sidak) of differences in MT between the three conditions revealed that the task was performed faster in sitting compared to both lying and provoking (both p < 0.001) conditions, but that there was no significant difference between lying and provoking conditions (p = 0.239). However, a significant interaction between group and condition (p = 0.032) confirmed that the change in MT across the successive conditions differed between healthy subjects and patients. Post hoc analysis showed that this mainly applied to the changes in MT from lying to provoking condition. In healthy subjects MT hardly increased from lying to provoking condition (Δ = 0.03 s; p = 0.998), while patients' MT did increase across the two conditions although the difference just failed to reach significance (Δ = 0.57 s; p = 0.088). However, the significant interaction between subgroup and condition (p = 0.042) makes clear that susceptible patients performed differently throughout the three conditions than unsusceptible patients did (Figure 4a). Patients in whom provocation was successful (susceptible) showed (further) increased MT in the provoking condition compared to lying condition, while no increase from lying to provoking condition was apparent in the patients unsusceptible to provocation.

As both subgroups showed similar accumulation of both baseline pain (p = 0.943) and experienced pain (p = 0.601) across conditions (Figure 4b), the worse motor performance in the provoking condition in the susceptible group is likely to be the result of successful provocation of pain-related cognitions (i.e. overprediction of pain).

Just as RT, MT did not significantly correlate with baseline fear of movement (TSK, r = - 0.187 to - 0.206, p > 0.5) nor catastrophizing (PCS, r = 0.127 to 0.143, p > 0.6) in any of the three task conditions. With respect to NRS scores there was only a significant moderate correlation between NRS_expected _and MT in sitting condition (r = 0.556; p = 0.044); all other correlations were rather low (r = 0.055 to 0.384) and non-significant (p > 0.15).

## Discussion

The goal of this study was to identify deviations in (general) motor performance of CLBP patients and determine in what way both pain experience and pain-related cognitions contributed to these deviations. For this, reaction time (RT) and movement time (MT) on a hand-function task were compared between the CLBP patients and healthy subjects in three increasingly provoking conditions. Results show that patients score higher on RT as well as MT compared to healthy subject, which implies that pain experience indeed can deteriorate motor performance in CLBP. Provoking pain-related cognitions affected only MT task performance at group level. Additional explorative analysis on individual data of the patient population identified two groups of patients based on susceptibility to provocation. The group of patients that was identified as being susceptible to provocation (n = 7) most clearly deviated from healthy MT performance throughout conditions. Correlation analysis showed that neither cognitions measured by means of baseline questionnaires (i.e. fear of movement and catastrophizing) nor pain (NRS scores) appeared to be related to either RT or MT.

### Reaction time

As hypothesized, RT of CLBP patients differed significantly from that of healthy subjects in all three conditions. This is in agreement with studies that found delayed muscle reaction times in CLBP patients as a responds to sudden (trunk) loading [16,17]. However, only a few other studies addressed pain patients' performance on an explicit motor RT task comparable to the one in the present study. Sjøgren et al. (2005) examined performance differences between chronic non-malignant pain patients and healthy controls on three neuropsychological tasks, including a simple reaction time task [18]. Their results demonstrated that the only difference in RT was seen when comparing the fastest patients with the fastest controls. Yet the heterogeneity of their study population (i.e. mixed chronic pain problems) might accord for the lack in difference between the majority of patients and healthy subjects. In contrast, Luoto et al. (1996) found a consistent yet non-significant trend of CLBP patients being slower than healthy subjects [19]. The significantly slower RTs of patients compared to healthy subjects found in the present study seem to support the results of Luoto and colleagues.

Apart from the significant group effect, results indicate that the condition in which the task was performed also influenced RT. RTs in lying and provoking conditions were higher than those in sitting condition. However, compared to healthy controls, patients showed no bigger increase from lying to provoking despite the apparent success of provoking pain-related cognitions by posture - as shown by the increased pain expectancy in the provoking condition. Based on this finding it can be concluded that the RT in CLBP patients was not influenced by cognitions.

This finding contradicts previous studies that demonstrated a deteriorating role for pain-related cognitions and RT performance [20-22]. Differences in experimental design (i.e. different RT tasks) may underlie this discrepancy.

### Movement time

As expected, CLBP patients showed slower movement times than healthy subjects in all conditions. This is in agreement with several other studies showing CLBP patients to be slower than healthy subjects on various low back motor tasks [3,17,23-26]. Yet to the authors' knowledge, the present study is the first that shows that this also applies to (general) motor tasks in neutral conditions as in the sitting condition CLBP patients appeared to perform the hand-function task substantially slower than healthy subjects. In a study with respect to a trunk positioning task Descarreaux et al. (2005) hypothesized that performing the task at lower speed (i.e. higher MT) might be an adapting motor control strategy used by patients aiming at less pain during task performance [24]. It is plausible that the difference between CLBP patients and healthy subjects in the present study is also based on such coping strategy. However, it should be noticed that other factors that differ between the patient and control group unidentified by the present study (e.g. differences in exercise habits or general physical activity) could have part in the differences seen. Hence, future studies with larger patient populations are needed to provide definitive evidence on the determinants of patients' worse motor performance, especially with respect to general motor tasks.

Apart from the significant differences between groups, results showed that the condition in which the task was performed affected MT. In line with the hypothesis, the MT appeared to deteriorate in lying and provoking condition compared to sitting condition. Apparently, the lying posture posed a challenge to task performance, since both patients and healthy subjects showed higher MT in lying condition compared to sitting condition. Subsequent performance in provoking condition did not further change MT in healthy subjects, while in patients MT seemed to increase. A significant interaction between group and condition proved that indeed patients performed differently throughout the conditions compared to healthy subjects. Presumably, this interaction is based on the trend for lower movement speed in the provoking condition in patients, which can be assumed to be attributable to the successful manipulation of pain-related cognitions (i.e. pain expectancy at least 1 NRS point higher than pain experience). The results of the patient subgroups analysis confirm this notion. Patients susceptible to provocation showed increased MT in provoking compared to lying condition, while patients unsusceptible to provocation showed no change in MT across these two conditions. In addition, the level of actual pain experience seemed to be lower in the susceptible group, indicating that pain (intensity) cannot explain the MT increase in provoking condition. This was further supported by the lack of correlation between NRS scores and MT. Neither can general cognition measures explain the increase as no correlations between baseline questionnaires (i.e. TSK and PCS) and MT were found and groups didn't differ with respect to these characteristics either. Thus, deteriorations in MT are assumedly determined by the amount of pain that patients anticipate rather than either the pain they actually experience or their general characteristics with respect to pain-related cognitions.

The trend that pain expectancy influences motor performance has previously been described by studies in which a deteriorating role of pain anticipation (i.e. increased expected pain) on motor performance was found. Al-Obaidi et al. (2000) showed that anticipation of pain was a strong predictor for variance in lumbar maximal isometric torque [23]. Also Pfingsten et al. (2001) found that pain anticipation played an important role in performance of a leg flexion task [11]. Yet the present study is the first to demonstrate the importance of pain anticipation for a motor task that does not directly involve the back. It is, however, possible that pain anticipation is not the only factor playing a role in the differences seen between the subgroups. Previous studies have shown patients' self-efficacy (i.e. their perceived ability to perform a task) to both influence task performance directly [27,28] and mediate the relation between pain-related cognitions and outcome [29,30]. One could argue that self-efficacy could have played a mediating role in the present results as well (i.e. susceptible patients being less confident regarding their performance and, thus, expecting more pain) and it would be interesting to look into the role of self-efficacy in future studies.

Still, the finding that posture can provoke falsely increased pain expectancy and, consequently, seems to worsen general motor task performance (i.e. MT) is considered to be of importance for CLBP therapy. For, it implies that adequate motor performance that is taught in a relatively safe clinical environment might worsen when patients are confronted with more challenging situations in daily life.

### Study limitations

Some remarks should be taken into account with respect to interpreting the results of the present study. First of all, the present study comprised a rather small study population and should, therefore, be regarded as a pilot study. In particular, the explorative results of the patient subgroups are based on small sample sizes. Additionally, the present study included only patients that were experiencing pain at the time of testing and, thus, one should be careful with extrapolating the results to the entire CLBP population. Future studies with larger patient populations are needed to confirm the preliminary findings of differences due to pain expectancy. Nevertheless, the fact that significant differences are found strongly strengthened the hypothesis that pain-related cognitions indeed influence motor performance of (symptomatic) CLBP patients.

Another possible limitation with respect to analysis is the lack of randomization of the three experimental conditions. Given that all conditions were more or less demanding on the back, the fixed order of condition might have caused fatigue to play a role in the last (i.e. provoking) condition, possibly more in CLBP patients than in healthy subjects. It was tried to avoid this by offering subjects rest periods between successive conditions, yet as fatigue was not measured during the experiments it was impossible to determine whether or not rest was sufficient. Still, to the authors opinion the possible influence of fatigue counts for little compared to the main reason to choose for task performance in the present fixed order (i.e. subsequently sitting, lying and provoking condition): to rule out that the effect of the provoking condition (i.e. increased pain-cognitions) sustained and would obscure performance measures in the other conditions.

Finally, a remark can be made on the choice of motor task. The hand-function task is minimally representative for daily life activities. It was performed in a laboratory situation and the task in itself did not represent a common motor activity. Therefore, one should be careful with translating the findings of the present study into a daily life situation. Nevertheless, the present study provides evidence for the role of pain and particularly pain-related cognitions with respect to motor performance and, therewith, offers a first indication of possible mechanisms behind the abnormal motor performance in CLBP patients. Future studies should further look into causalities with respect to chronic pain and performance, since identifying the main determinants of deviated motor performance in low back pain provides valuable information for motor therapy improvement. Furthermore, confirming these results in a design with activities of daily living should be an aim in future studies, because effect of pain and pain-related cognitions for relevant motor activities is probably of value for therapy improvement.

## Conclusion

The present study shows that CLBP patients have worse motor task performance compared to healthy subjects. This might be partly attributable to an altered motor strategy due to pain experience. Yet the worsening of performance in case of successful additional provocation of pain-related cognitions (i.e. unreal pain expectancy, without increase in actual pain experience) indicates that pain-related cognitions might be as important as mere pain experience.

## Competing interests

The authors declare that they have no competing interests.

## Authors' contributions

DK: Design, conducting the experiment, analysis of the data, writing and editing of the manuscript. MV: Initiating the study, design, monitoring progress and editing of the manuscript. HH: Design, monitoring progress and editing of the manuscript. All authors read and approved the manuscript.

## Pre-publication history

The pre-publication history for this paper can be accessed here:

http://www.biomedcentral.com/1471-2474/12/211/prepub
